# Anthelmintic Treatment and the Stability of Parasite Distribution in Ruminants

**DOI:** 10.3390/ani13111882

**Published:** 2023-06-05

**Authors:** Eric R. Morgan, Anne Segonds-Pichon, Hubert Ferté, Patrick Duncan, Jacques Cabaret

**Affiliations:** 1 School of Biological Sciences, Queen’s University Belfast, 19, Chlorine Gardens, Belfast BT9 5DL, UK; 2 Babraham Institute, Cambridge CB22 3AT, UK; anne.segonds-pichon@babraham.ac.uk; 3 Faculté de Pharmacie, Université de Reims Champagne-Ardenne, SFR Cap Santé, EA7510 ESCAPE–USC VECPAR, 51 rue Cognacq-Jay, 51096 Reims, France; hubert.ferte@univ-reims.fr; 4 Centre d’Etudes Biologiques de Chize, CNRS UPR 1934, 79360 Beauvoir-sur-Niort, France; patrick.duncan3b@gmail.com; 5 ISP, INRAE, Université Tours, UMR1282, 37380 Nouzilly, France; jcabaret37@gmail.com

**Keywords:** parasite, aggregation, overdispersion, mechanism, Taylor’s power law, metapopulation dynamics, immunity, regulation, complex systems, fluctuation scaling

## Abstract

**Simple Summary:**

Parasites tend to be unevenly distributed among hosts, with most hosts in a population carrying few parasites and most of the parasites found in a few heavily infected individuals. This property, known as aggregation or overdispersion, is important to the diagnosis of parasite infections in groups of animals and their management. Analysis of 325 sets of gastrointestinal nematode parasite counts from wild and domestic ruminants, some including worms from post-mortem examinations and others faecal egg counts, explored how overdispersion changed in relation to various factors. A systematic relationship was found between the variance in parasite counts and their means, in accordance with the previously demonstrated Taylor’s power law. Furthermore, groups of livestock frequently treated with anthelmintics had more aggregated parasite burdens. For parasite species that stimulate strong immunity, aggregation was lower for faecal egg counts than for adult worm burdens. Considered together, the results suggest that immunity to parasites tends to stabilise distributions and that treatment interferes with this process and leads to greater clustering of infections among individuals. Understanding the processes that drive parasite aggregation will help to manage them in farmed systems and more generally could shed light on how animal distributions are shaped in changing environments.

**Abstract:**

Parasites are generally overdispersed among their hosts, with far-reaching implications for their population dynamics and control. The factors determining parasite overdispersion have long been debated. In particular, stochastic parasite acquisition and individual host variation in density-dependent regulation through acquired host immunity have been identified as key factors, but their relative roles and possible interactions have seen little empirical exploration in parasite populations. Here, Taylor’s power law is applied to test the hypothesis that periodic parasite removal destabilises the host-parasite relationship and increases variance in parasite burden around the mean. The slope of the power relationship was compared by analysis of covariance among 325 nematode populations in wild and domestic ruminants, exploiting that domestic ruminants are often routinely treated against parasite infections. In *Haemonchus* spp. and *Trichostrongylus axei* in domestic livestock, the slope increased with the frequency of anthelmintic treatment, supporting this hypothesis. In *Nematodirus* spp., against which acquired immunity is known to be strong, the slope was significantly greater in post-mortem worm burden data than in faecal egg counts, while this relationship did not hold for the less immunogenic genus *Marshallagia*. Considered together, these findings suggest that immunity acting through an exposure-dependent reduction in parasite fecundity stabilises variance in faecal egg counts, reducing overdispersion, and that periodic anthelmintic treatment interferes with this process and increases overdispersion. The results have implications for the diagnosis and control of parasitic infections in domestic animals, which are complicated by overdispersion, and for our understanding of parasite distribution in free-living wildlife. Parasite-host systems, in which treatment and immunity effectively mimic metapopulation processes of patch extinction and density dependence, could also yield general insights into the spatio-temporal stability of animal distributions.

## 1. Introduction

Complex systems consist of interacting elements, which participate in dynamic processes. This relationship often takes the form ‘fluctuations = constant × average^α^’ [1]. This power law was first discovered by Fairfield Smith [2], who devised a formula for the yield of crop fields, and it was further developed in ecology by Taylor [3]. The same relationship has been explored for the dynamics of complex networks and has been termed fluctuation scaling. It has been applied with success in many fields of research [1], including physics, climatology, social sciences, and life sciences, with the value of α ranging from one to two.

In parasitology, it has long been appreciated that helminth infections are typically aggregated (or equivalently overdispersed), with the majority of worms in the minority of animals [4,5], and this case is typical of fluctuation scaling. Aggregation has an important influence on host–parasite population interactions [6,7], the effects of chemotherapy on group morbidity [8], parasite transmission [9], diagnosis [10], and selection for drug resistance [11]. Overdispersion also provides an opportunity for targeted control of parasites since selective elimination of the heaviest burdens will disproportionately reduce the parasite population [12]. Relative to repeated whole-group treatments, this approach could delay the development of anthelmintic resistance by preserving refugia for drug-susceptible genotypes [13,14].

The importance of aggregation to parasite dynamics and control throws into stark relief the long-running debate over what generates aggregation in parasite populations. A number of mechanisms have been proposed and some shown to be capable of generating realistic levels of aggregation in experimental or model systems [15,16,17]. However, the relative importance of different mechanisms and interactions between them have yet to be satisfactorily elucidated in less constrained populations. Thus, for example, theoretical work has shown that immunity per se, by acting on parasite infrapopulations in a density-dependent manner, tends to decrease aggregation [6]. However, variation between hosts in the extent to which they express immunity, for example, through genetic differences, increases aggregation [18]. Host immunity could therefore play an important role in generating patterns of parasite distribution in many animal systems, but its effect will depend on the strength and form of the immune response, as well as its variability [19]. Since acquired immunity also depends on individual levels of exposure, heterogeneity in parasite acquisition could modify its expression and effect at the population level. Heterogeneous parasite acquisition can be caused inter alia by spatial variation in the distribution of parasite infective stages [20] and the effects of climatic stochasticity on infective stage development and survival [21], both of which are factors that have been proposed to have strong influences on parasite distribution independently of host immunity. Heinzmann et al. [22] showed that stochastic parasite acquisition can explain natural levels of aggregation in tapeworms without recourse to host variation in immunity, whereas aggregation of gill flukes in fish was explained by host factors and not variation in exposure [17]. It is of course possible, even likely, that the balance of factors underlying parasite distribution varies between systems and that no universal answer exists to the question of what drives parasite aggregation. Nevertheless, better understanding of aggregation is needed to support control efforts, especially of nematodes in grazing ruminants, in which anthelmintic resistance threatens food production and security [23], demanding more creative approaches to control [24].

Gastrointestinal nematode infections in ruminants provide a useful model for parasite aggregation. A range of closely related host and parasite species allow for the investigation of factors of interest without major confounding effects of taxonomy or gross variations in body size or life history [25]. Large amounts of data can be harvested from populations worldwide from post-mortem worm counts and indirect measures of parasite burden, such as faecal egg counts. Most species share a similar life cycle, producing eggs that pass with the faeces and develop to the infective third stage, before being ingested with herbage and maturing to adult parasites. The development and survival of the free-living stages are highly dependent on ambient conditions, especially temperature and moisture [21]. Given adequate exposure, hosts generally develop partial protective immunity, which manifests as age-resistance, although its rate and extent vary with the parasite and host species, as well as individual capacity. Host immunity acts to suppress parasite establishment, fecundity, and survival [26]. Most domestic ruminants are routinely treated with anthelmintic drugs to maintain productivity, but the frequency of such treatments varies widely between and within farming systems. This fact constitutes a natural experiment in that treatment will effectively obliterate infrapopulations, disrupting processes that act to stabilise host-parasite interactions and increasing the stochasticity of the host experience of parasites.

We exploit this situation using Taylor’s power law to explore patterns of aggregation in trichostrongyloid nematodes of grazing ruminants. Using Taylor’s law, we seek to integrate this study into developing ideas in macroecology. Thus, host–parasite systems are effectively metapopulations, with host immunity resembling density-dependent processes in free-living animals and host treatment mimicking patch extinctions [27]. Interactions between environmental stochasticity and density dependence are potentially central to the dynamics of free-living animals, as well as parasite populations [28]. The ruminant–nematode system might therefore be a fruitful arena in which to explore the contrasting effects of stabilising and destabilising ecological factors on patterns of animal distribution. We are particularly interested in whether increased stochasticity leads to increases in the slope parameter of Taylor’s power law, as proposed by Morand and Krasnov [29], and in how host immunity and the disruptive effect of treatment might interact to generate patterns of parasite aggregation, altering the variance around the mean relationship.

## 2. Materials and Methods

Data from 325 separate host–parasite combinations were analysed, drawn from published studies, as well as additional data held by the authors on natural, tracer, and experimental studies (Table 1). Datasets measured the intensity of parasite infection, either through faecal egg counts or post mortem worm counts. The average size of the host group sampled was 29 (range 9–123), and all but 24 of the groups included at least 20 individuals. Host systems consisted of sheep, goats, cattle, and saiga antelopes (*Saiga tatarica*) in Kazakhstan; sheep in Poland, France, and Morocco; goats in France and Martinique; cattle in Argentina, Brazil, and New Zealand; and roe deer (*Capreolus capreolus*) in France. Parasites were all from the superfamily Trichostrongyloidea in Clade V of Blaxter’s molecular phylogeny [30], with the exception of *Trichuris capreoli* in Clade I. Genera included *Haemonchus*, *Ostertagia*, *Marshallagia*, *Teladorsagia*, *Trichostrongylus* and *Nematodirus*. Each parasite taxon–host group combination was considered independent, which obscures possible interactions between parasites within individual hosts, but it was accepted for this group level analysis.

Most data were available as post-mortem worm counts, in which adult nematodes are recovered from the gastrointestinal tract after slaughter. There was therefore no opportunity for repeated sampling. Indirect measures of parasite burden were available for some groups through faecal nematode egg counts. These counts generally provide a reasonable reflection of adult worm burdens [35], but they are modified by immunity, which acts to suppress worm fecundity. The treatment history of the groups varied considerably: wild ruminants were not treated at all, and livestock was treated with anthelmintics between zero and five times per year. To some extent, this difference reflected geographical differences in parasite challenges and farming systems, and treatment frequency was confounded by host species. Animals were mostly kept permanently on pastures through the grazing season, giving some opportunity for the host–parasite relationship to stabilise before slaughter. A few groups were tracer animals on experimental plots, which had grazed for a limited time before slaughter, with little opportunity to develop a stable nematode infra-community.

The degree of parasite aggregation (=overdispersion) was assessed by estimating the exponent of Taylor’s power law using loglinear regression of variance and means across host-parasite groups. Thus:

Log *V* = *a* + *b*·Log *M* (1)

where *M* is the arithmetic mean parasite count within the host group, *V* is its variance, and *a* and *b* are fitted parameters. The slope of the regression, *b*, corresponds to Taylor’s exponent, and a higher value indicates a more strongly aggregated parasite distribution. This approach largely circumvents the limitations of methods that make a priori assumptions about the shape of parasite distributions, such as the negative binomial distribution (NBD), in which measures of parasite aggregation are sensitive to changes in mean abundance, complicating comparisons between host groups and systems [36,37]. Fitting of Equation (1) to estimate aggregation requires data from many host groups but has the advantage of providing an objective comparison of the contributions of different host and parasite characteristics to the overall variance.

After assessing the overall fit of Taylor’s power law to the combined data, the slope parameter *b* was compared across different groups using analysis of covariance (ANCOVA). Starting with a fully saturated model, factors that were not significant were excluded in turn, the least significant first, to leave a parsimonious model of factors affecting the slope. Because variation in most factors was very unevenly spread between groups, those factors proposed a priori to have a strong effect on the host–parasite relationship were selected for further investigation within host subsets. These factors were host immunity and the frequency of anthelmintic treatment. No independent information was available at the level of host immunity. However, since one of the earliest and strongest effects of immunity to gastrointestinal nematodes in ruminants is suppression of egg output [38], differences in slope between post-mortem worm count and faecal egg count data should reflect immunity. This relationship is difficult to investigate across the trichostrongylids because eggs of different species are morphologically similar, so faecal egg counts cannot be related to any particular nematode species or genus. However, eggs of *Marshallagia* (Trichostrongylidae) and *Nematodirus* (Moleinidae) can be differentiated from the others listed above, so these genera were selected for this analysis. Treatment is predicted to disrupt the host–parasite relationship by hindering stabilisation through development of density dependence and host immunity. More frequent treatment should represent a source of stochasticity since newly acquired burdens will be subject to spatiotemporal variation in infective stage availability while having less time to reach a steady state through host regulation.

## 3. Results

### 3.1. Overall Fit to Taylor’s Power Law

**Figure 1:**
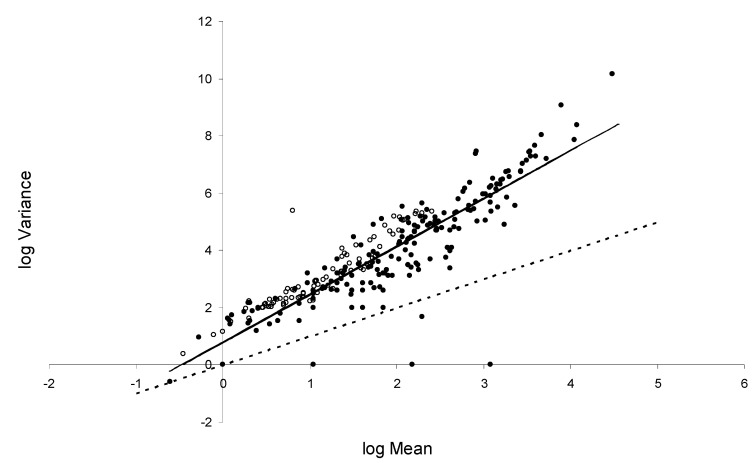
Regression of log_10_ (variance) on log_10_ (mean parasite abundance) for the entire dataset (*n* = 325 groups). Solid line: regression equation (log *V* = 0.781 + 1.683 log *M*); dashed line: log *V* = log *M*. Open circles: faecal egg counts; filled circles: post mortem worm counts.

Across the whole dataset, log *M* was positively correlated with log *V* (*r*^2^ = 0.821, *p* < 0.001). Linear regression of log *V* on log *M* was highly significant (MS regression 941.215, MS residual 0.633, *F*_1,323_ = 1486.04, *p* < 0.001), with estimated coefficients *a* = 0.781 (95% confidence intervals 0.609–0.952) and *b* = 1.683 (1.597–1.769). Model fit to the entire data set is shown in Figure 1. ANCOVA using all the factors in Table 1 revealed that only host sex (*F*_2,274_ = 3.811, *p* = 0.023) and frequency of treatment (*F*_2,274_ = 36.678, *p* < 0.001) significantly affected regression slope *b*. However, both factors were confounded with host species. Hence, free-ranging wildlife, and sheep in Kazakhstan were not treated, whereas other hosts were treated and goats especially frequently, while the most intensively treated groups tended to be of mixed sex, which was allocated a third sex category. When considered across all host groups, parasite taxa did not significantly affect the regression slope, although intercept varied more widely (Table 2).

### 3.2. Immunity: Nematodirus and Marshallagia in Sheep and Antelopes

**Figure 2:**
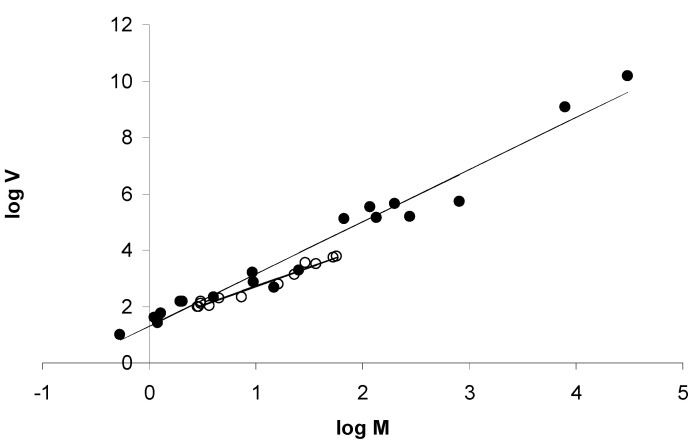
Effect of life cycle stage on regression slope *b* for *Nematodirus* spp. Coefficients: post mortem worm counts (filled circles) *a =* 1.296 (95% CI 0.983–1.608), *b* = 1.854 (1.690–2.018); faecal egg counts (open circles) *a* = 1.355 (1.176–1.534), *b* = 1.356 (1.197–1.515).

*Nematodirus* from the abomasum and small intestine of sheep (*n* = 10 groups) and saiga antelopes, *Saiga tatarica* (*n* = 12 groups), in Kazakhstan were included, comprising several different species of which *N. gazellae* and *N. oiratianus* were most abundant. Host groupings differed also by age (less than one year, 11; more than one year, 11), sex (male, 7; female, 15), and method of parasite enumeration (post-mortem counts of adult worms, 11; faecal egg counts, 11). None of the animals were treated for nematode infection. Only the method of parasite enumeration had a significant effect on slope (*F*_1,17_ = 10.339, *p* = 0.003), with fitted slope parameter *b* being higher for worm counts than for faecal egg counts (Figure 2).

*Marshallagia marshalli* from sheep (*n* = 9 groups) and saiga antelopes [9] in Kazakhstan differed by age (less than one year old = 6, older = 11), sex (male = 6, female = 12) and method of parasite enumeration (post-mortem worm counts = 8, faecal egg counts = 10). None of these factors affected the mean–variance relationship, which was described by a simple loglinear regression with coefficients *a* = 1.077 (95% CI 0.796, 1.358) and *b* = 1.937 (1.736, 2.137): *F*_1,19_ = 409.29, *p* < 0.001. The slope of the regression was closer to that for adult worm counts of *Nematodirus* in the same hosts than to egg counts of *Nematodirus*, which had a lower slope parameter *b*.

### 3.3. Frequency of Treatment: Haemonchus and Trichostrongylus in Lambs and Goats

The effect of the reported annual number of treatments on regression slope was investigated in domestic ruminants naturally infected with *Trichostrongylus axei* and *Haemonchus contortus*.

**Figure 3:**
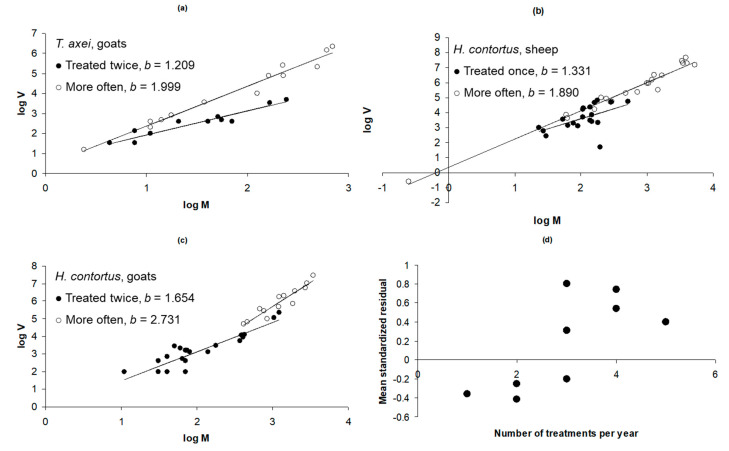
Effect of treatment frequency on Taylor’s parameter *b* (regression slope, Equation (1)). (**a**) *Trichostrongylus axei* in goats. Coefficients: *a* = 0.713 (95% CI 0.484, 0.942), *b* = 1.209 (1.041, 1.377), *F*_1,16_ = 8.678, *p* < 0.001 (twice, filled circles); Coefficients: *a* = 0.364 (−0.079, 0.806), *b* = 1.999 (1.777, 2.222), *F*_1,11_ = 390.11, *p* < 0.001 (more often, open circles). (**b**) *Haemonchus contortus* in sheep, coefficients *a* = 0.934 (95% CI −1.154, 3.023), *b* = 1.331 (0.314, 2.348), *F*_1,18_ = 7.557, *p* = 0.013 (treated once annually, filled circles); *a* = 0.343 (95% CI −0.082, 0.768), *b* = 1.890 (1.741, 2.040), *F*_1,20_ = 693.52, *p* < 0.001 (treated frequently, or tracer, open circles). (**c**) *Haemonchus contortus* in goats. Coefficients: *a* = −0.181 (95% CI −0.874, 0.511), *b* = 1.654 (1.316, 1.991), *F*_1,22_ = 103.46, *p* < 0.001 (treated twice annually, filled circles); *a* = −2.501 (95% CI −4.505, −0.498), *b* = 2.731 (2.086, 3.375), *F*_1,11_ = 86.97, *p* < 0.001 (treated three or four times annually, open circles). (**d**) Correlation between residuals on host-parasite specific regression and number of treatments for *T. axei* in goats and for *Haemonchus* spp. in sheep, goats and cattle (*r^2^* = 0.53, *n* = 9, *p* = 0.026; Spearman’s *ρ* = 0.74, *p* = 0.022).

The generalist species *Trichostrongylus axei* was found in sheep, goats, cattle, and deer. However, only groups of goats differed in treatment frequency (twice annually = 18, three times = 4, four times = 9), also varying by climate (temperate = 22, tropical = 9) and age (less than one year old = 10, older = 21). ANCOVA identified the number of treatments as significantly affecting slope (*F*_2,27_ = 17.599, *p* < 0.001) (Figure 3a). Across all host species (*n* = 61 groups), number of treatments was found to significantly affect slope (*F*_4,57_ = 13.612, *p* < 0.001), although treatment frequency was strongly confounded with host species (Table 3).

In 42 groups of lambs in which *H. contortus* burdens were counted post mortem, the factors recorded were climate (temperate *n* = 32, tropical *n* = 10) and the annual frequency of treatments (once *n* = 20, five times *n* = 10). Some groups of animals (*n* = 12) were kept as tracers. All animals were less than one year of age and of mixed or unknown sex. Analysis of covariance initially identified that treatment category significantly influenced slope, but the difference was only significant between hosts treated annually and the other categories (five times or tracers). The sample was therefore split into two categories of treatment: once annually (*n* = 20), representing hosts in which host–parasite interactions had some opportunity to reach a steady state; and naïve or frequently treated hosts (*n* = 22), in which this opportunity was much diminished. The effect of treatment category on regression slope was highly significant (ANCOVA MS 2.896, *F*_1,39_ = 9.575, *p* = 0.004), with the variance being higher at a given mean in hosts treated more frequently (Figure 3b).

A total of 37 groups of goats naturally infected with *H. contortus* were analysed, varying by climate (temperate *n* = 28, tropical *n* = 9), age (less than one year of age *n* = 12, older *n* = 25), and frequency of treatment (twice annually *n* = 24, three times *n* = 4, four times *n* = 9). Neither climate nor age significantly affected the slope of the regression, and these factors were excluded from the analysis. The final model again included the frequency of treatment as the only significant influence on the slope of the regression. Separate regressions for goats treated three or four times did not yield significantly different slopes, and these groups were combined for visual clarity (Figure 3c). Both the mean and the variance increased with the frequency of treatment, and at a given mean, the variance was greater in groups treated more often (ANCOVA MS 2.624, *F*_2,33_ = 7.875, *p* = 0.002). Cattle infected with *H. placei* were included in the analysis, but the sample size was too small (13 groups: temperate *n* = 5, tropical *n* = 8; permanent *n* = 6, tracer *n* = 7) to detect any effect of covariates on regression slope.

When *Haemonchus* spp. burdens were analysed across all host categories excluding tracers, host species (*F*_2,65_ = 8.807, *p* < 0.001) and treatment frequency (*F*_4,65_ = 7.256, *p* < 0.001) influenced the regression slope, with climate and age having no effect, although adults were present only among goats. The interaction between host and treatment frequency was not significant. The slope was lower in sheep (1.331) than in goats (1.654) or cattle (1.675). The mean of the standardised residuals correlated significantly with the number of treatments across host–parasite systems (Figure 3d).

## 4. Discussion

Analysis of the loglinear regression of variance on mean parasite abundance was used to investigate the factors determining the exponent (slope) of Taylor’s power relationship and hence parasite aggregation in 325 supra-populations of trichostrongyloid nematodes in ruminants. A value for the slope of one could correspond to two main scenarios: either events (i.e., successive infections) are independent, or the time scale of the survey is so short that two events (two infections) cannot happen within the same time window. A value for the slope of two may occur when events (infections) are identical and synchronic or when there is a universal distribution of infections within the infrapopulation of hosts, which only varies by multiplicative factors. These scenario proposals are interpreted from Eisler et al., 2008 [1] theoretical formalism, which is not explicitly related to biology. A value of one does not correspond to what is known about helminth infections in ruminants since there is no independence of successive infections. Infection occurs by means of ingesting herbage that is contaminated with larvae, so if the chance of an infection event is high, another is more likely. At the same time, the time window for infection was large enough in our data to permit successive reinfections. A value of two may not correspond fully either, as events (infections) are not necessarily identical, but this value may be related to synchronic infections, which can arise as a result of seasonality. It could also correspond to the existence of a multiplicative factor, such as genetic susceptibility or the influence of treatments. We further detail our expectations concerning the influence of these factors on aggregation and the slope of Taylor’s power relationship below. The slope of the overall regression of 1.683 and the tendency of individual regression slopes to cluster between 1.5 and 2 suggest a mixture of random and multiplicative processes. A value for the slope of one is compatible with a Poisson distribution of parasites within host suprapopulations, while a slope of two indicates logarithmic processes. A slope of 1.5 is compatible with the negative binomial distribution (NBD). The slope of Taylor’s relationship can therefore be used to design appropriate transformations for stabilising variance in parasite data for statistical analysis (Boag 1992 [39]) or to select distribution-specific models. Our finding of slopes generally close to, but slightly greater than, this value suggest that the NBD is a broadly appropriate model for parasite distributions in this system, although overdispersion above this level might sometimes call for modifications, such as zero inflation (Denwood et al., 2008 [40]). While the previous work cited above described statistical distributions of parasites in animal populations, including grazing ruminant livestock, there have been only very limited attempts to model this distribution across multiple host populations or to relate observed patterns to unifying theories, such as fluctuation scaling.

The a priori expectation was that acquired immunity would have a dominant effect, tending to stabilise host-parasite interactions and decrease levels of aggregation, although the analysis was conducted independently of this assumption. With only cross-sectional data available, the effect of immune-mediated regulation of parasite burdens was initially assessed by comparing the slope of Taylor’s power relationship between *Nematodirus* worm burdens and faecal egg counts. *Nematodirus* spp. are relatively immunogenic among the trichostrongyloids (Israf et al., 1997 [41]), with burdens highest in young animals and generally declining in grazing ruminants older than one year of age, even at low levels of challenge (Boag and Thomas, 1977 [42]). Previous work showed that there is a good correlation between adult *Nematodirus* spp. burdens and faecal egg counts in ruminants younger than one year of age in Kazakhstan but not in animals older than one year of age (Morgan et al. 2005 [31]). This finding strongly suggests that protective immunity acts to decrease parasite fecundity. A similar analysis of *Marshallagia* spp. in the same system showed a good correlation between worm burden and faecal egg counts across all ages. The present analysis showed that the slope was lower for faecal egg count data than for adult parasite counts in *Nematodirus* but not in *Marshallagia*, suggesting that host immunity, insofar as it acts through parasite fecundity, on balance decreases parasite aggregation. This finding is in line with previous studies that ascribed a predominantly stabilising influence to host immunity, (e.g., [19]).

Boag et al. [43] also proposed that host immunity played a prominent role in determining parasite distributions and noted dynamic variations in the slope parameter among natural infections of gastrointestinal helminths in rabbits. That parasite aggregation was generally lower in rabbits showing signs of infection with the immunosuppressive myxoma virus suggested that the overall effect of immunity was to increase parasite aggregation, in contrast with the present results. However, in Boag’s study, the observed results could equally be due to the preferential loss of high parasite burdens through the death of hosts co-infected with the virus, directly or through increased coinfections [44], which would indeed decrease nematode aggregation. Parasite-induced mortality is rarely an important player in the dynamics of gastrointestinal nematode infection in domestic ruminants, so we argue that our data provide stronger evidence for a stabilising effect of immunity on parasite aggregation.

In *Haemonchus* spp. and *Trichostrongylus axei* in domestic livestock, the slope increased with the frequency of anthelmintic treatment. This relationship held across livestock species and was consistent within the most sampled species. Given the previous finding that immunity tends to stabilise parasite variance, the most logical explanation is that frequent treatment, by removing parasite burdens that are then replenished from pasture larvae, effectively postpones the development of stable host–parasite associations and consequently increases parasite aggregation.

The global analysis confirmed that parasite taxa, at least within the broadly similar trichostrongyloid superfamily, do not have a dominant effect on parasite aggregation. Certainly, the present analysis does not support previous suggestions that Taylor’s slope parameter *b* is a stable characteristic of parasite species [3,39,45].

Given the apparent importance of host protective immunity in determining parasite aggregation, it is perhaps surprising that age did not emerge as a significant factor in any of the analyses. However, this finding could be explained by the correlation between parasite challenge and treatment frequency. Thus, a high level of parasite challenge would favour the build-up of immunity with age (and hence stability) but also more frequent treatment (countering stability). Climate was also found to not be a significant predictor of Taylor’s slope parameter, despite its known strong effects on infective larval availability [21]. Thus, greater climatic stochasticity in arid or tropical-oceanic regions might be expected to act to destabilise host–parasite interactions by increasing the unpredictability of parasite acquisition. This outcome did not materialise, perhaps due to the smoothing of seasonal effects over time, with parasite burden representing accumulated infection.

Taylor’s power law has previously been used to investigate determinants of spatio-temporal variance in the abundance of free-living animals, (e.g., [46,47,48]). Host–parasite systems offer some advantages of tractability since the sampling unit, a point of debate in studies of animal distribution, is necessarily that of the individual host. Furthermore, host treatment offers a pseudo-experimental way of destabilising the system through removal and recolonisation of habitat patches to an extent that is rarely possible in free-living systems. Studies of parasite aggregation can therefore contribute to understanding the stability of animal distributions in metapopulations. From the present results, it might be hypothesised that more disrupted metapopulations would remain more aggregated due to lesser opportunity for the action of stabilising density-dependent constraints.

The present results have implications for parasite control. Treatment and interaction with host immunity appear to have strong effects on parasite aggregation. Disruption of stabilising host–parasite interactions could explain increased aggregation of human soil-transmitted helminths following frequent mass treatment [49,50]. Control strategies that rely on aggregation, such as targeted selective treatment of individuals carrying high parasite burdens [12], might therefore be easier to apply to disturbed than to stable host–parasite systems or, for example, earlier in the grazing season before stabilisation occurs. Targeted treatment itself is likely to lead to reduced parasite aggregation through removal of the highest burdens, thus making further rounds of selective treatment less efficient or harder to apply. On the other hand, a case could be made that differences between parasite and host species in degree of aggregation are rather less important than might be supposed and that intra-system factors, such as protective immunity, are more important, so strategies based on assumed levels of aggregation should be quite transferable between systems. This case might only be true within coherent host–parasite groupings, such as that in the present study. There is still a need for broader descriptive analyses of typical levels of aggregation in differently managed, as opposed to free-living, animal species (c.f., [36,37]). Experimental studies might also be designed to test the hypotheses raised here, for example, by tracking parasite aggregation over time and in response to anthelmintic interventions.

Fluctuation scaling as a phenomenon is not restricted to specific systems [1,51], although mechanisms and outcomes vary across them, yielding insights into diverse biological interactions, including infectious disease dynamics [52,53] and forest growth [54,55]. Further application of the concept to nematodes in livestock [56,57], including through Taylor’s power law, could potentially help with problems such as altered population dynamics, diagnosis, and sustainable interventions, as future management necessarily moves beyond routine chemical suppression as a core management strategy [24,58].

## 5. Conclusions

In conclusion, the results showed that the level of aggregation of helminth parasites among ruminant livestock hosts was predicted by loglinear regression of the variance against the mean, as described by Taylor’s power law. The slope of this relationship clustered around 1.6, consistent with the negative binomial distribution, and it varied between systems. Higher parasite aggregation in populations subject to more frequent antiparasitic treatments suggests a destabilising effect of such treatments, perhaps through inhibition of density-dependent processes, especially host immunity. Better understanding of fluctuation scaling in this and related systems could help to guide sustainable control strategies and inform broader ecological theories.

## Figures and Tables

**Table 1:** Host groups sampled. *n* = number of groups in each category. Host groups sampled. Some data from saiga antelopes, cattle, and sheep were previously published (saiga: Morgan et al., 2005, 2006 [31,32]; sheep: Nowosad et al., 2003 [33]; goats: Gasnier et al., 1997 [34]). Abbreviations: Climate: Temp = Temperate, Trop = Tropical; Age: Juv = Juvenile, Ad = Adult, Mx = Mixed or not known; Sex: M = Male; F = Female; Method: PM = Post mortem, FEC = Faecal egg count; Parasite: Other = Other trichostrongylids; Treatment: N/yr = Number per year; Tracer: Perm = Permanent; Trac = Tracer; Expmtl = Experimental infection.

Factor	Host		Climate		Age		Sex		Method		Parasite		Treatment	Tracer	
Categories (*n*)	5		3		3		3		2		5		5	3	
	Sheep	129	Temp	202	Juv	180	M	42	PM	234	*Haemonchus*	98	N/yr	Perm	289
	Goat	81	Trop	36	Ad	128	F	74	FEC	91	*T. axei*	91		Trac	33
	Cattle	22	Arid	87	Mix	17	Mix	209			*Marshallagia*	25		Expmtl	3
	Deer	66									Other trichostrongylids	33			
	Saiga	27									*Trichuris*	51			

**Table 2:** Regression coefficients of Taylor’s Power Law (Equation (1)) for different parasite taxa across all host groups. CI = confidence interval.

Taxon	Intercept, *a*	95% CI	Slope, *b*	95% CI
*Haemonchus*	−0.515	−0.955–−0.075	2.057	1.884–2.229
*T. axei*	0.172	−0.056–0.400	1.956	1.833–2.079
*Marshallagia*	1.077	0.796–1.358	1.937	1.736–2.137
*Trichuris*	0.987	0.791–1.184	1.886	1.763–2.010
*Nematodirus*	1.130	0.887–1.373	1.847	1.699–1.995
Other trichostrongyles	1.058	0.759–1.357	1.750	1.508–1.993

**Table 3:** Regression coefficients of Taylor’s Power Law, Equation (1), for groups of hosts infected with *T. axei* and treated with differing frequencies.

Treatment Frequency	0	1	2	3	4
Host species	Deer	Sheep	Goats	Goats	Goats, cattle
*a* (intercept)	1.305	0.215	0.713	0.375	0.529
*b* (slope)	1.517	1.970	1.209	2.029	1.889

## Data Availability

The data presented in this study are partially available on request from the corresponding author. The data are not publicly available due to restrictions on the sharing of original individual parasite data generated by third parties.
